# The Dynamic Scleral Extracellular Matrix Alterations in Chronic Ocular Hypertension Model of Rats

**DOI:** 10.3389/fphys.2020.00682

**Published:** 2020-07-03

**Authors:** Chen Qiu, Jing Yao, Xi Zhang, Rong Zhang, Xinghuai Sun, Shaohong Qian

**Affiliations:** ^1^Department of Ophthalmology and Vision Science, Eye and Ear, Nose, Throat Hospital, Shanghai Medical College, Fudan University, Shanghai, China; ^2^NHC Key Laboratory of Myopia, Fudan University, Shanghai, China; ^3^Laboratory of Myopia, Chinese Academy of Medical Sciences, Shanghai, China; ^4^Department of Ophthalmology, Zhongshan Hospital of Fudan University, Shanghai, China; ^5^State Key Laboratory of Medical Neurobiology, Institutes of Brain Science, Fudan University, Shanghai, China; ^6^Shanghai Key Laboratory of Visual Impairment and Restoration, Fudan University, Shanghai, China

**Keywords:** sclera, extracellular matrix, matrix metalloproteinase 2, intraocular pressure, glaucoma

## Abstract

Intraocular pressure (IOP) generates stress and strains in the laminar cribrosa and sclera, which may affect the development and progression of glaucoma. Scleral stiffness and material components have changed under elevated IOP. However, the detailed changes of the components of the hypertensive sclera are not well understood. In this study, we aimed to investigate the changes of the main components in the scleral extracellular matrix (ECM), and matrix metalloproteinase 2 (MMP2) and their relationship with time under chronic elevated IOP in Sprague–Dawley rats. An ocular hypertension model was established in the right eyes by anterior chamber injection with 0.3% carbomer solution. The left eye was used as the contralateral control. Immunofluorescent imaging of the tissue frozen sections, Western blot analysis, and quantitative PCR (qPCR) were performed to detect the expressions of type I collagen (COL1), elastin, and MMP2 in the sclera. The ocular hypertension model was successfully established. As compared to the left eyes, the immunofluorescence imaging, Western blot analysis, and qPCR showed that COL1, elastin, and MMP2 were significantly increased in the right eyes at 1 week (all *P* < 0.05). At 2 weeks, COL1 in the right eyes tended to be lower than that in the left eyes, while elastin and MMP2 were still higher (all *P* < 0.05) in the right eyes. When the IOP was elevated for 4 weeks, both COL1 and MMP2 were lower than those in the left eyes (all *P* < 0.05), while elastin between the two eyes was similar (*P* > 0.05). Under this 4-week hypertensive state, COL1 and elastin were initially elevated at 1 week, and then obviously reduced from 2 to 4 weeks. Consistently, MMP2 was gradually increased, with a peak at 2 weeks, and then decreased at 4 weeks. In conclusion, the chronic elevated IOP induced dynamic scleral ECM alterations in rats in a pressure- and time-dependent manner. MMP2 may play an important role in the balance between ECM synthesis and degradation and could potentially be a novel target for glaucoma intervention.

## Introduction

Glaucoma is the leading cause of irreversible blindness across the world, and the most efficient treatment is sufficient intraocular pressure (IOP) reduction. The apoptosis of retinal ganglion cells (RGCs) is the feature of glaucomatous damage, with a key site at the optic nerve head (ONH). IOP causes mechanical load (Sigal et al., 2004; Girard et al., 2011; Coudrillier et al., 2012; Nguyen et al., 2013) and generates stress and strain in the sclera, which are magnified at the ONH (Sigal et al., 2005; Girard et al., 2009; Grytz et al., 2011; Norman et al., 2011; Ivers et al., 2016). The properties of the sclera may affect glaucoma progression. Myopic eyes, with reduced scleral stiffness (McBrien et al., 2009), may have a slower glaucoma progression rate as compared to non-myopic eyes (Sawada et al., 2018; Han et al., 2020). The aged sclera exhibits an accumulated extracellular matrix (ECM) and increased stiffness (Coudrillier et al., 2015; Nguyen et al., 2018), which is more likely to have deterioration of glaucoma (De Moraes et al., 2012; Bommakanti et al., 2020). Additionally, the mechanical strain may alter the biomechanical properties of the sclera, which may indirectly affect the apoptosis of RGCs.

According to the biomechanical studies in the past decade, a glaucomatous sclera (especially the peripapillary sclera) is stiffer than the normal sclera in postmortem human eyes, as measured by inflation tests (Coudrillier et al., 2012). Girard et al. (2011) studied the finite element models of monkey eyes and reported that the scleral stiffness increased after exposure to elevated IOP, as detected by speckle interferometry. Inflation testing of mice’s posterior sclera also revealed stiffer pressure–strain responses in chronic glaucomatous eyes, mimicking the findings in monkey and human glaucomatous eyes (Nguyen et al., 2013). In addition, the glaucomatous eyes had slower circumferential creep rates in the peripapillary sclera than do the normal eyes (Coudrillier et al., 2012).

Corresponding to the mechanical changes, scleral remodeling is also observed under elevated IOP. Increased fibrous components and decreased non-fibrous components have been indicated in the sclera of a mouse ocular hypertension model (Nguyen et al., 2013). The scleral permeability of mice may decrease with chronic IOP elevation (Pease et al., 2014). New technologies such as small-angle light scattering (Zhang et al., 2015) and wide-angle X-ray scattering (Pijanka et al., 2014) have also shown changes of the scleral structures induced by an elevated IOP. Oglesby et al. (2016) analyzed the scleral protein expressions in experimental mouse glaucoma using liquid chromatography coupled with tandem mass spectrometry (LC-MS/MS) and found proliferating scleral fibroblasts and altered protein expressions in the sclera. It is speculated that the strain-related scleral remodeling could in turn regulate tissue biomechanics, which may exert a crucial effect in the development and progression of the disease. Generally, scleral remodeling is a dynamic, complex process implying an imbalance between ECM synthesis and degradation, but its detailed cellular and molecular changes have not been well documented. The sclera is mainly composed of type I collagen (COL1), matrix metalloproteinases (MMPs), and tissue inhibitor of matrix metalloproteinases (TIMPs), with additional elastin fibrils and proteoglycans. So far, most studies of scleral remodeling have concentrated on the exploration of myopia. Active scleral remodeling plays a critical role during the process of myopia development. Scleral alterations in myopia mainly involve ECM degradation, with different expressions of MMPs (Rada and Brenza, 1995), TIMPs (Rada et al., 1999), collagen fibrils (Gentle et al., 2003), and non-fibrous components (Moring et al., 2007). MMP2 is one of the most important MMPs during the myopia process. The production of MMP2 was demonstrated to increase in animal models of myopia, enhancing the degradation of the scleral ECM components such as COL1 (Liu et al., 2017; Zhao et al., 2018). Concordantly, biomechanical tests of the myopic models showed an increased creep rate and elasticity of the sclera (Phillips et al., 2000; McBrien et al., 2009). Interfering with the expression of MMP2 could slow down myopia development in animal models (Liu et al., 2017; Zhao et al., 2018).

Interestingly, mechanical stretching could alter the expressions of MMPs, and/or TIMP2 in scleral fibroblasts (Yamaoka et al., 2001; Fujikura et al., 2002; Shelton and Rada, 2007). However, the role of MMP2 in the remodeling of hypertensive sclera *in vivo* has not been elucidated yet. In the present study, dynamic alterations of the main components in the scleral ECM (COL1, elastin, and MMP2) were investigated in a chronic ocular hypertension model of rats.

## Materials and Methods

All the animal protocols and procedures were in accordance with the Association for Research in Vision and Ophthalmology Statement for the Use of Animals in Ophthalmic and Vision Research. The protocols were approved by the Institutional Review Board and Ethics Committee of Eye and Ear, Nose, Throat Hospital of Fudan University.

### Experimental Chronic Ocular Hypertension Model in Rats

Sprague–Dawley rats were fed in standard cages under a 12-h light/dark cycle. An experimental chronic ocular hypertension model was made in rats (males, 240–250 *g*) by anterior chamber injection with 0.3% carbomer solution (Carbomer 940 polymer, Solarbio, Shanghai, China), as previously described elsewhere (Xu et al., 2002; Kim et al., 2013). Simply, rats were anesthetized with an intraperitoneal injection of 10% chloral hydrate (3 ml/kg; Macklin, Shanghai, China). A 33-gage needle (Kindly, Shanghai, China) was used to puncture near the corneal limbus. After penetrating into the anterior chamber, a 33-gage syringe (Hamilton, Ghiroda, Romania) was then used to inject 20 μl of 0.3% carbomer solution into the anterior chamber. Antibiotic ointment (ofloxacin; Shengyang Xingqi Pharmaceutical Co., Ltd, China) was applied after the procedure.

The injected eyes with an IOP 5 mmHg higher than the contralateral eyes after injection were considered successful ocular hypertension (Chan et al., 2007). Repeated injection of the carbomer solution was performed once the IOP declined below the successful level. Samples were collected at 1 (*n* = 28), 2 (*n* = 34), and 4 weeks (*n* = 29) after the models were successfully established, and their use for experiments is shown in Table 1. At the indicated time, the rats were humanely euthanized by an overdose of anesthesia (600 mg/kg chloral hydrate). The posterior sclera was our focus of investigation due to its important role shown in the biomechanical tests (Coudrillier et al., 2012).

**TABLE 1:** Number of eyes used for the experiments.

	**1-week group**	**2-week group**	**4-week group**
**Application**	**(*n* = 28)**	**(*n* = 34)**	**(*n* = 29)**
TUNEL assay	4	4	5
Retrograded labeling of RGCs	5	5	5
Immunohistochemical staining	5	7	5
Western blot	8	11	8
RT-qPCR	6	7	6

**TUNEL*, terminal deoxynucleotidyl transferase-mediated dUTP nick-end labeling; *RGC*, retinal ganglion cell; and *RT-qPCR*, reverse transcription quantitative polymerase chain reaction.*

### IOP Measurements

Intraocular pressure was measured in anesthetized rats at the same time of day (from 10 a.m. to 12 a.m.) using a rebound tonometer (TonoLab, ICare, Espoo, Finland) by the same operator. IOP measurements were performed at baseline (before anterior chamber injection) and twice a week after the models were made. For each eye’s measurement, the average value of 10 independent readings of machine-generated IOP was calculated. IOP values were represented as the mean ± standard deviation (SD).

### Preparation of Frozen Sections

After anesthetization, the rats were treated with cardiac perfusion using 4% formaldehyde phosphate-buffered saline (PBS). The enucleated eyes were fixed in 4% formaldehyde PBS at 4°C overnight. After dehydration in 20% and 30% sucrose solutions consecutively, the anterior segments were removed, and the tissue blocks containing the posterior eyeballs were then embedded with optimal cutting temperature compound (Tissue-Tek, Sakura Finetek Inc., Tokyo, Japan) and frozen at −80°C. Eight-micrometer slices were cut along the sagittal axis with the freezing microtome, air dried, and stored at −30°C until use.

### TUNEL Assay

The apoptotic cell in the retina frozen sections was initially measured by the terminal deoxynucleotidyl transferase-mediated dUTP nick-end labeling (TUNEL) assay. Using the In Situ Cell Death Detection kit, TMR red (Roche, IN, United States), the TUNEL assay was performed following the manufacturer’s instructions. Hoechst 33342 (Life Technologies, NY, United States) was employed for nuclei staining. Images were captured by confocal fluorescence microscopy (Leica SP8, Berlin, Germany). The setting parameters of the confocal fluorescence microscopy (including the speed, zoom factor, pixel size, smart gain value, and pinhole value) remained the same to ensure that the images were taken under the same laser intensity and image acquisition. Retinas approximately two to three disk diameters from the optic nerve were captured and analyzed. The percentages of positive staining cells were calculated using ImageJ software (National Institutes of Health, Bethesda, MD, United States).

### Retrograded Labeling of RGCs

Retrograde labeling of RGCs was conducted in rats 7 days before the indicated time. After anesthetization, 3% FluoroGold dissolved in 10% dimethyl sulfoxide (Sigma-Aldrich, MO, United States) was gently injected into the bilateral superior colliculi of the rats, with 2 μl per injection, as described previously (Dai et al., 2011). At the indicated time, the eyeballs were removed, fixed, and the whole retinas were carefully dissected and processed in flat-mount preparations. Images were taken using confocal fluorescence microscopy.

### Immunohistochemical Staining

To determine the expressions of COL1, α-elastin, and MMP2 in the posterior pole of the sclera, frozen tissues were analyzed using immunofluorescence staining. Briefly, frozen sections were rinsed with PBS twice and permeabilized with 1 mM HCl at room temperature for 45 min. After washing with PBS three times, the frozen sections were blocked with 5% bovine serum albumin (BSA) for 1 h and then incubated at 4°C overnight with primary antibodies: anti-COL1 (1:200), anti-MMP2 (1:100; Novus, CO, United States), and anti-α-elastin (1:100; Abcam, Cambridge, United Kingdom). A secondary antibody (1:200; Alexa 488, Thermo Fisher Scientific, Waltham, MA, United States) was applied at room temperature for 1 h after PBS immersion. Nuclei were stained by Hoechst 33342 for 15 min. Images were captured by confocal fluorescence microscopy at the posterior sclera two to three disk diameters from the optic nerve. Semi-quantitative analysis was performed by ImageJ.

### Western Blot

Western blot was used to further quantify the expressions of COL1, α-elastin, and MMP2 in the posterior sclera. Individual rat protein was prepared using the Tissue Protein Extraction Kit containing phenylmethanesulfonyl fluoride (PMSF; ComWin Biotech, Beijing, China), which was designated to extract the secreted extracellular proteins. The proteins were boiled with a sodium dodecyl sulfate polyacrylamide gel electrophoresis (SDS-PAGE) sample buffer (Takara, Dalian, China) at 96°C for 10 min. Each sample containing 25 μg total proteins underwent electrophoresis on 10% SDS-PAGE gels and was then electrotransferred to a nitrocellulose blotting membrane at 300 mA for 90 min. Membranes were blocked with 5% nonfat dry milk at room temperature for 1 h and then incubated with diluted primary antibodies at 4°C overnight. The antibodies used for Western blot were commercially purchased as follows: rabbit polyclonal anti-GAPDH (1:10,000; Bioworld, St. Louis Park, MN, United States), rabbit polyclonal anti-collagen type I (1:1,000), rabbit polyclonal anti-alpha-elastin (1:500), and rabbit polyclonal anti-MMP2 (1:1,000). GAPDH was kept stable under mechanical strain (Blaauboer et al., 2011; Qu et al., 2015) and was used as the loading control. After rinsing with 1X Tris–buffered saline/0.05% Tween 20 (TBST) for 30 min, the membranes were incubated with a peroxidase-conjugated secondary antibody (1:3,000; Cell Signaling Technology, Boston, MA, United States) at room temperature for 1 h. Chemiluminescence (Super-Signal^TM^ West Femto Substrate Trial Kit, ThermoFisher, MA, United States) was used when capturing images. The immunoreactive bands were analyzed in triplicate with ImageJ software.

### Reverse Transcription qPCR

The total RNA of the rat sclera samples were extracted using a TRIzol reagent (Invitrogen, CA, United States) and reverse transcribed to cDNA using the PrimeScript^TM^ RT Reagent Kit (Takara, Dalian, China). Quantitative PCR (qPCR) was subsequently conducted with SYBR Premix Ex Taq^TM^ (Takara, Dalian, China) on the ABI ViiA7 Real-Time PCR System (Thermo Lifetech, CA, United States). The following conditions were used for qPCR: 95°C for 30 s, then 50 cycles of 95°C for 5 s, and 60°C for 30 s. The primer sequences were as follows: *GAPDH* (housekeeping gene): forward 5′-ACGGCAAGTTCAACGGCACAG-3′ and reverse 5′-CGACATACTCAGCACCAGCATCAC-3′; *COL1*: forward 5′;-TGTGCGATGGCGTGCTATGC-3′ and reverse 5′-TGCGTCTGGTGATACATATTCTTCTGG-3′; *Elastin*: forward 5′-GCCCTGGGATATCAAGGTGG-3′ and reverse 5′-CACTG GCCTGTTGTCCCC-3′; *MMP2*: forward 5′-ACACCAAGAACT TCCGACTATCCAATG-3′ and reverse 5′-CAGTACCAGT GTCAGTATCAGCATCAG-3′.

### Statistical Analysis

Statistical analyses were performed using SPSS software (version 19.0, SPSS, Inc., Chicago, IL, United States). Student’s *t* test or one-way ANOVA was used, with *P* value < 0.05 considered statistically significant.

## Results

### IOP Elevation and RGC Change in a Chronic Ocular Hypertension Model of Rats

**FIGURE 1:**
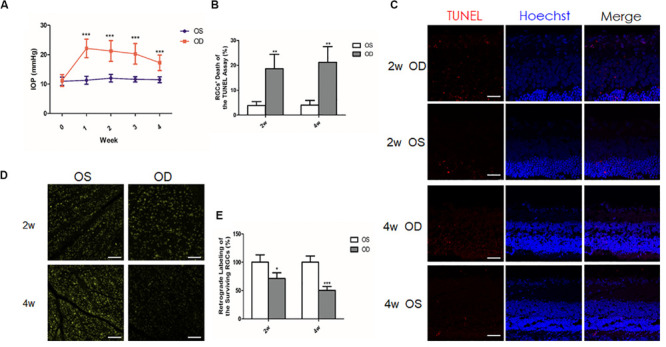
Intraocular pressure (IOP) elevation and retinal ganglion cells (RGCs) change in a chronic ocular hypertension model of rats. **(A)** Mean IOPs of the hypertensive right eyes and the contralateral left eyes after anterior chamber injection of 0.3% carbomer solution. **(B)** Quantitative analysis of the percentage of the apoptotic RGCs of the terminal deoxynucleotidyl transferase-mediated dUTP nick-end labeling (TUNEL) assay. **(C)** TUNEL assay showed increased apoptotic (*red*) RGCs in the hypertensive right eyes (*OD*) when compared to the contralateral left eyes (*OS*; *n* = 4 for each group). Nuclei were stained with Hoechst (*blue*). **(D)** Retrograde labeling of the surviving RGCs revealed less RGCs in the hypertensive right eyes than in the left eyes (*n* = 5 for each group). **(E)** Quantitative analysis of retrograde labeling. Data were expressed as the mean ± SD. *P < 0.05; **0.001 < *P* < 0.01; and ***P < 0.001. *Scale bar*, 50 μm.

To ensure the successful establishment of an ocular hypertension model in rats, we measured IOP twice a week. Repeated injection of the carbomer solution was performed in 12 rats when the IOP dropped down from the successful level (5 mmHg higher than baseline; Chan et al., 2007). Among them, 4 out of 12 rats were excluded as the IOP was below this level after repeated injections. Finally, 1-week (*n* = 28), 2-week (*n* = 34), and 4-week (*n* = 29) ocular hypertension models were successfully established. The IOPs of the successful models were calculated at different time points (Figure 1A). Before anterior chamber injection, the mean IOPs of the right eyes (OD; 11.32 ± 1.4 mmHg), and the left eyes (OS; 10.81 ± 1.42 mmHg) were similar (*P* = 0.592). After injection, the mean IOPs of the hypertensive right eyes were significantly elevated than those of the contralateral left eyes at every time point (1-week group: 22.12 ± 3.17 *vs*. 10.5 ± 1.38 mmHg; 2-week group: 21.25 ± 3.54 *vs*. 11.25 ± 1.41 mmHg; and 4-week group: 17.25 ± 2.65 *vs*. 10.91 ± 1.38 mmHg, all *P* < 0.001).

To investigate the influence of IOP elevation on RGCs, TUNEL analysis, and retrograde labeling of the RGCs were used to detect dead or live RGCs, respectively. After the model was successfully established, TUNEL assay of the frozen sections showed more apoptotic RGCs in the right eyes than in the left eyes (4.02 fold change at 2 weeks, *n* = 4, *P* = 0.006; 6.29 fold change at 4 weeks, *n* = 5, *P* = 0.005; Figures 1B,C). Consistently, retrograde labeling of the RGCs revealed a significant decrease of the remaining RGCs in the right eyes (approximately 28.7% loss at 2 weeks, *n* = 5, and *P* = 0.013; 49.6% loss at 4 weeks, *n* = 5, and *P* < 0.001) when compared with the left eyes (Figures 1D,E).

### ECM Alterations of the Sclera Under Chronic Elevated IOP for 1 Week

**FIGURE 2:**
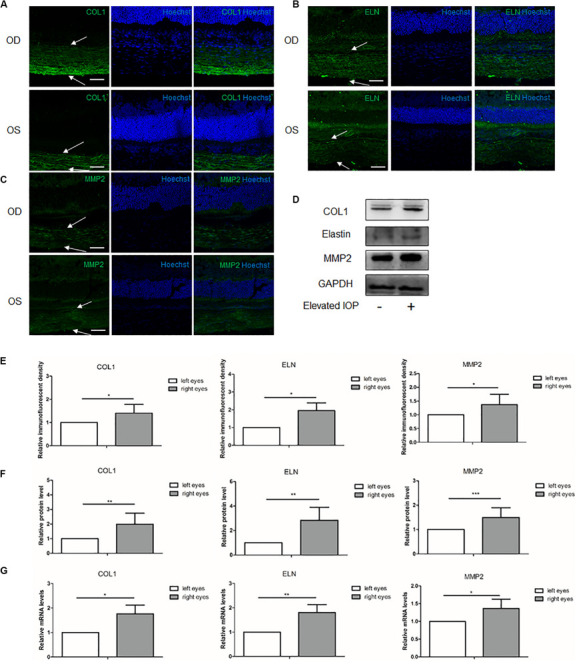
Expressions of the scleral extracellular matrix (ECM) components under chronic elevated intraocular pressure (IOP) for 1 week. The ocular hypertension model was established for 1 week in rats. Immunohistochemical staining showed the scleral **(A)** collagen I (*green*), **(B)** elastin (*green*), and **(C)** matrix metalloproteinase 2 (MMP2, *green*) expressions in the hypertensive right eyes (*OD*) and the contralateral left eyes (*OS*). *White arrows* indicate the sclera. *Scale bar*, 50 μm. **(D)** Immunoreactive bands of collagen I, elastin, and MMP2 in the contralateral left eyes and the hypertensive right eyes. **(E)** Semi-quantitative analysis of the immunofluorescent images (*n* = 5). Quantification of the protein expressions **(F**; *n* = 8) and mRNA levels **(G**; *n* = 6) of collagen I, elastin, and MMP2. GAPDH was set as the housekeeping gene. Data were demonstrated as the mean ± SD of three replicates. *P < 0.05; **0.001 < *P* < 0.01; and ***P < 0.001.

To investigate the influence of the elevated IOP on the main ECM components and the MMP2 in the posterior sclera, the expressions of these components were compared between the hypertensive right eyes (OD) and the contralateral left eyes (OS) in eight separate rat samples. At 1 week, the immunoreactivities of COL1, elastin, and MMP2 were all higher in the right eyes than in the left eyes (Figures 2A–C,E). Western blot and qPCR analyses further validated the protein and mRNA expressions of these components, respectively. The protein levels of COL1, elastin, and MMP2 in the right eyes were upregulated 1.99 (*P* = 0.002), 2.84 (*P* = 0.001), and 1.49 (*P* < 0.001) fold changes, respectively, (Figures 2D,F). The transcriptional levels of COL1 (1.76 fold change, *P* = 0.032), elastin (1.81 fold change, *P* = 0.004), and MMP2 (1.36 fold change, *P* = 0.021) were concordantly increased in the hypertensive right eyes (Figure 2G).

### ECM Alterations of the Sclera Under Chronic Elevated IOP for 2 Weeks

**FIGURE 3:**
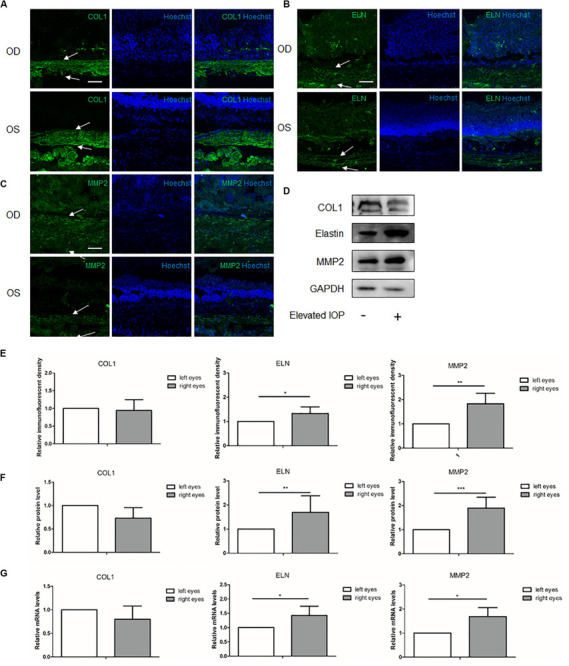
Expressions of the scleral extracellular matrix (ECM) components under chronic elevated intraocular pressure (IOP) for 2 weeks. The ocular hypertension model was established for 2 weeks in rats. Immunohistochemical staining showed the scleral **(A)** collagen I (*green*), **(B)** elastin (*green*), and **(C)** matrix metalloproteinase 2 (MMP2, *green*) expressions in the hypertensive right eyes (*OD*) and the contralateral left eyes (*OS*). **(E)** Semi-quantitative analysis (*n* = 7). *White arrows* indicate the sclera. *Scale bar*, 50 μm. **(D)** Western blot and **(F)** the quantification analysis showed the protein expressions of collagen I, elastin, and MMP2 (*n* = 11). **(G)** mRNA expressions of collagen I, elastin, and MMP2 (*n* = 7). GAPDH was set as the loading control. Data were expressed as the mean ± SD of three replicates. *P < 0.05; **0.001 < *P* < 0.01; and ***P < 0.001.

At 2 weeks, immunohistochemical staining showed that the immunoreactivity of COL1 in the hypertensive right eyes (OD) seemed lower than that in the contralateral left eyes (OS), but the semi-quantitative analysis of the immunofluorescent density did not reach statistical significance (Figure 3A). The immunoreactivities of elastin and MMP2 were still upregulated in the right eyes (Figures 3B,C,E). Western blot analysis revealed a decreased COL1 with borderline significance (0.73 fold change, *P* = 0.078) and increased elastin and MMP2 (elastin: 1.70 fold change, *P* = 0.003; MMP2: 1.89 fold change, *P* < 0.001) when compared to the left eyes (Figures 3D,F). The results of the qPCR (COL1: 0.80 fold change, *P* = 0.204; elastin: 1.46 fold change, *P* = 0.023; and MMP2: 1.69 fold change, *P* = 0.027) were consistent with those of the Western blot (Figure 3G). The 2-week group exhibited an alleviated upregulation of the main ECM components as compared to the 1-week group.

### ECM Alterations of the Sclera Under Chronic Elevated IOP for 4 Weeks

**FIGURE 4:**
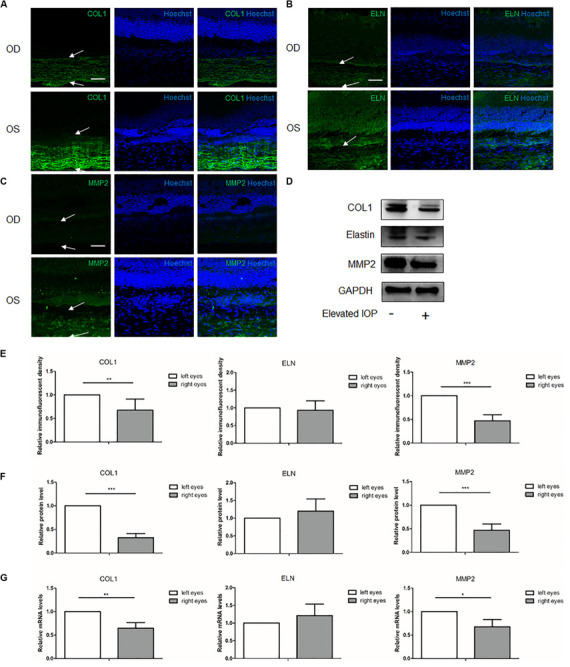
Expressions of the scleral extracellular matrix (ECM) components under chronic elevated intraocular pressure (IOP) for 4 weeks. The ocular hypertension model was established for 4 weeks in rats. Immunohistochemical staining showed the scleral **(A)** collagen I (*green*), **(B)** elastin (*green*), and **(C)** matrix metalloproteinase 2 (MMP2, *green*) expressions in the hypertensive right eyes (*OD*) and the contralateral left eyes (*OS*). *White arrows* indicate the sclera. *Scale bar*, 50 μm. **(E)** Semi-quantitative analysis of the immunofluorescent images (*n* = 5). **(D)** Western blot and **(F)** the quantification analysis showed the protein expressions of collagen I, elastin, and MMP2 (*n* = 8). **(G)** mRNA levels of collagen I, elastin, and MMP2 (*n* = 6). GAPDH was set as the loading control. Data were expressed as the mean ± SD of three replicates. *P < 0.05; **0.001 < *P* < 0.01; and ***P < 0.001.

At 4 weeks, the immunofluorescent imaging, Western blot, and qPCR analysis revealed that COL1 and MMP2 were dramatically decreased (Figures 4A–G). Semi-quantitative analysis of the Western blot showed 0.33 (*P* < 0.001) and 0.47 (*P* < 0.001) fold changes of the COL1 and MMP2, respectively, in the hypertensive right eyes (OD) as compared to the contralateral left eyes (OS). At the transcriptional levels, the qPCR revealed 0.64 (*P* = 0.005) and 0.68 (*P* = 0.015) fold changes of the COL1 and MMP2, respectively. With respect to elastin, the protein (1.20 fold change, *P* = 0.515) and mRNA (1.21 fold change, *P* = 0.352) expressions in the right eyes were similar to those in the left eyes (Figures 4F,G).

### Trend of Scleral ECM Alterations

**FIGURE 5:**
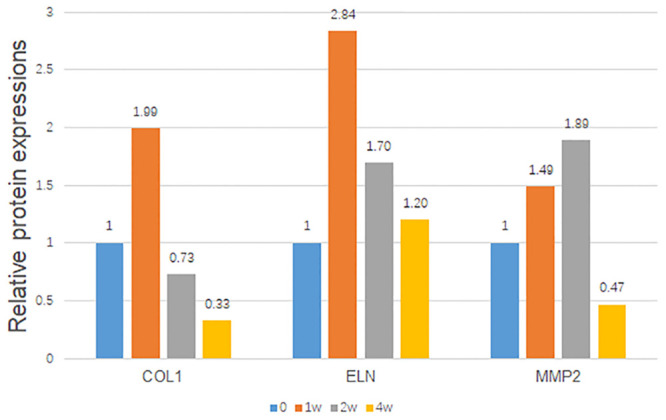
Trends of the relative scleral protein expressions with time. The protein/GAPDH values of the hypertensive right eyes to the contralateral left eyes were demonstrated with time. GAPDH was set as the loading control.

After the anterior chamber injection, the trends of scleral ECM alterations under the elevated IOP with time are shown in Figure 5, suggesting dynamic protein changes in a pressure- and time-dependent manner. COL1 and elastin were initially elevated, and then obviously reduced from 2 to 4 weeks. MMP2 was gradually increased, with a peak at 2 weeks, and then decreased at 4 weeks.

## Discussion

In this study, we successfully established the chronic ocular hypertension model in rats by anterior chamber injection with 0.3% carbomer solution. Under elevated IOP, the main components in the scleral ECM and MMP2 demonstrated dynamic alterations with time, implying that MMP2 may be involved in the balance of ECM degradation and synthesis.

The sclera forms the outer layer of the eyeball and endures more strain than the retina and choroid (Wu et al., 1987). Scholars have confirmed that the sclera undergoes complicated, dynamic changes during eye development, the aging process, myopia, and IOP changes (Bisplinghoff et al., 2009; Coudrillier et al., 2012). The complex scleral alterations may involve changes in the scleral components, stiffness, axial length, and so on. The change of scleral stiffness coincided with the process of scleral ECM remodeling. In myopic eyes, the ECM of the sclera degraded rather than synthesized, and scleral stiffness decreased. The fibrous and non-fibrous components reduced, and the viscoelasticity of the sclera increased in animal models of myopia (Norton and Rada, 1995; Phillips et al., 2000; Gentle et al., 2003; Moring et al., 2007). However, the stiffness of the hypertensive sclera increased, and the compliance/viscoelasticity decreased in the early stage of the chronic ocular hypertension models (Girard et al., 2011; Coudrillier et al., 2012; Nguyen et al., 2013). Consistently, our study demonstrated more synthesis than degradation in the early stage. Increased COL1 and elastin may thicken and stiffen the sclera, weaken the scleral compliance, and alter the biomechanics of the ONH.

However, more ECM degradation was initiated in the later stage in our study. COL1 and elastin were gradually decreased with time. The expression of COL1 at 2 and 4 weeks was even lower than that at baseline. These results indicated that short-term, mild-to-moderately elevated IOP might stimulate a compensatory effect in the sclera to sustain the elevated IOP, manifesting more synthesis than degradation, while long-term stimulation of the elevated IOP may exceed the compensatory limit of the sclera, leading to the degradation of the ECM components with a gradual increase of MMP2.

Collagen fibrils constitute more than 50% of the sclera and crucially affect its biomechanical properties (Keeley et al., 1984). Approximately 90% of the scleral dry weight comes from COL1 (Watson and Young, 2004). COL1 was eminently reduced during the development of myopia (Norton and Rada, 1995; Gentle et al., 2003), especially in its early stage. Changes of the other types of collagen fibrils were not as apparent as those of COL1. The preferential loss of COL1 compared to type I and type II collagen was also observed in the sclera of myopic eyes (Gentle et al., 2003). Differently from these studies, COL1 was obviously increased in the early stage of our ocular hypertension model. At 2 weeks, the fibrils started to decrease with an accumulative effect of the elevated IOP.

Elastin is another component detected in the sclera which is associated with its viscoelasticity. In the acute ocular hypertensive model, elastin took immediate reaction to resist the elevated IOP (Geraghty et al., 2012). Similarly, elastin was upregulated dramatically at 1 week in our study, which possibly suggested a protective mechanism that may limit scleral canal expansion under an elevated IOP. However, sustained elevated IOP downregulated the elastin to baseline. We speculated that it might be a homeostatic response of the sclera to the elevated IOP.

Several studies have reported that the mechanical strain applied to scleral fibroblasts can trigger the release of MMPs and TIMPs (Yamaoka et al., 2001; Fujikura et al., 2002; Cui et al., 2004; Shelton and Rada, 2007), which may then lead to a remodeling of the scleral ECM. MMPs are secreted as latent proenzymes that are capable of degrading ECM substrates, almost all the proteins. MMP2 is one of the classic and most studied proteins. It has been reported to be collagenolytic and elastolytic. MMP2 could digest the solubilized monomers of COL1 by interacting with the collagen fibrils (Aimes and Quigley, 1995). For instance, in the early stage of skin wound healing, MMP2 may prolong the wound closure by lowering collagen, including the COL1 (Kanno et al., 2019). The upregulation of MMP2 in the sclera during the myopia process contributed to the degradation of the collagen and elongation of the eye length (Rada and Brenza, 1995; Rada et al., 1999). Reducing scleral MMP2 could alleviate the reduction of COL1 accumulation and retard myopia progression in mice (Zhao et al., 2018). Elastin is usually resistant to proteolysis. However, the 72-kDa gelatinase (MMP2) was capable of degrading insoluble elastin when combined with fibronectin type II-like repeats (Shipley et al., 1996). Inhibition of MMP2 significantly stimulated elastin expression in airway fibroblasts from patients with allergic asthma when compared to those from normal controls (Ingram et al., 2016). The role of MMP2 in modulating scleral elastin has not been well investigated. However, our data revealed a potential relationship between MMP2 and elastin in the hypertensive sclera, which requires further investigation.

The present study also showed an increased level of MMP2 in the early stage of ocular hypertension, which may be triggered by ocular hypertension (Karamichos et al., 2008; Huisman et al., 2016). Despite the increased degradation of ECM induced by MMP2, the levels of COL1 and elastin were still upregulated, indicating more synthesis than degradation of the ECM in the early stage. The increased levels of the ECM components may continuously trigger MMP2 secretion and degrade more ECM components. As a result, the production of these ECM components began to decline at 2 weeks with gradually increasing MMP2. Further, dramatically reduced levels of the ECM components may, in turn, terminate the MMP2 production in the late stage. It might be speculated from our results that MMP2 may be a key point to maintain the balance between ECM synthesis and degradation during ocular hypertension. Further studies are needed to elucidate the role of MMP2 in scleral remodeling, which might bring up new therapeutic avenues to prevent the progression of glaucoma.

## Conclusion

In summary, our study demonstrated that chronic elevated IOPs in rats could induce dynamic scleral ECM changes in a pressure- and time-dependent manner. MMP2 may play a critical role in these changes. Interventions to modulate the scleral ECM remodeling may provide a new approach for glaucoma therapy, and MMP2 may be used as a potential novel therapeutic target.

## Data Availability Statement

All datasets generated for this study are included in the article/**Supplementary Material**.

## Ethics Statement

The animal study was reviewed and approved by Institutional Review Board and Ethics Committee of Eye and Ear, Nose, Throat Hospital of Fudan University.

## Author Contributions

CQ, XS, and SQ designed the study. CQ and RZ performed the experiments. CQ and XZ analyzed the data. CQ and JY wrote the manuscript. CQ, JY, and SQ revised the manuscript. All authors contributed to the article and approved the submitted version.

## Conflict of Interest

The authors declare that the research was conducted in the absence of any commercial or financial relationships that could be construed as a potential conflict of interest.
